# Activation of the TCA Cycle to Provide Immune Protection in Zebrafish Immunized by High Magnesium-Prepared *Vibrio alginolyticus* Vaccine

**DOI:** 10.3389/fimmu.2021.739591

**Published:** 2021-12-07

**Authors:** Jun Yang, Xiao-li Yang, Yu-bin Su, Xuan-xian Peng, Hui Li

**Affiliations:** ^1^ Center for Proteomics and Metabolomics, State Key Laboratory of Bio-Control, Southern Marine Science and Engineering Guangdong Laboratory (Zhuhai), School of Life Sciences, Sun Yat-sen University, Guangzhou, China; ^2^ Laboratory for Marine Fisheries Science and Food Production Processes, Qingdao National Laboratory for Marine Science and Technology, Qingdao, China; ^3^ Key Laboratory of Functional Protein Research of Guangdong Higher Education Institutes, Department of Biotechnology, College of Life Science and Technology, Jinan University, Guangzhou, China

**Keywords:** *Vibrio alginolyticus*, live vaccine, metabolomics, TCA cycle, innate immunity

## Abstract

Vaccines are safe and efficient in controlling bacterial diseases in the aquaculture industry and are in line with green farming. The present study develops a previously unreported approach to prepare a live-attenuated *V. alginolyticus* vaccine by culturing bacteria in a high concentration of magnesium to attenuate bacterial virulence. Furthermore, metabolomes of zebrafish immunized with the live-attenuated vaccines were compared with those of survival and dying zebrafish infected by *V. alginolyticus*. The enhanced TCA cycle and increased fumarate were identified as the most key metabolic pathways and the crucial biomarker of vaccine-mediated and survival fish, respectively. Exogenous fumarate promoted expression of *il1β*, *il8*, *il21*, *nf-κb*, and lysozyme in a dose-dependent manner. Among the five innate immune genes, the elevated *il1β*, *il8*, and *lysozyme* are overlapped in the vaccine-immunized zebrafish and the survival from the infection. These findings highlight a way in development of vaccines and exploration of the underlying mechanisms.

## Introduction

Aquaculture contributes significantly to mankind, not only providing huge amounts of high-quality food, but also promoting human economic development. However, the rapid intensification of aquaculture has adverse ramifications, such as disease outbreaks, which are a major impediment to the growth of aquaculture (1, 2). *Vibrio alginolyticus* is an important pathogen to both aquatic animals and humans. In aquaculture, it infects a variety of aquatic animals, such as orange-spotted grouper (3), large yellow croaker (4), and white shrimp (5, 6), leading to massive mortality of farmed fishes and thereby causing serious damage to aquaculture. In humans, it causes sepsis, gastroenteritis, wound, and ear infections. Antibiotics are effective to control infection caused by the bacterium, but the misuse and abuse of antibiotics negatively impacts on aquatic environments, food safety, and development of antibiotic resistance (7, 8). Therefore, an alternative approach is especially needed.

Vaccines, taking advantages of reducing dependence on antibiotics, are known to be used to provide long-lasting protection against diseases and thus improve fish health and reduce disease outbreaks with no drug residues. Several types of vaccines have already been developed, such as inactivated whole-cell (9, 10), live attenuated vaccines (9, 11–13), protein subunits (14, 15), anti-idiotypic (16), and DNA vaccines (17, 18). To date, most commercially available and authorized vaccines used in the aquaculture industry are the inactivated whole-cell vaccines, whereas the development of live-attenuated and combined vaccines is in a demanding process (19). Comparatively, the inactivated whole-cell vaccines usually provide poor but stable immunity, while live attenuated vaccines have higher protective efficacy and they do not require adjuvants that only require single or few injections to provide protection. In Europe, a licensed live-attenuated vaccine against *V. vulnificus* protecting eels from vibriosis has been issued in clinic (20), suggesting live-attenuated vaccine is an effective approach to control infection caused by *Vibrio* spp. However, the development of vaccines is still limited in aquaculture due to the large reservoir of bacterial pathogens in the ecosystem that can hardly be protected by a single type of vaccine and the various types of species of aquatic animals. Thus, a novel vaccine-developing strategy is urgently needed.

Live-attenuated vaccines are developed in several ways. Random mutation(s) were generated through successive passage of virulent strains under specific conditions (21–23) or transposon based random insertion mutations or allelic exchange have been frequently adopted (24, 25). Among the targeted genes, genes encoding metabolic enzymes are selected for vaccine candidates. The mutation of isocitrate dehydrogenase (*icd*) gene in *Vibrio anguillarum* attenuate bacterial virulence and subsequently protected rainbow trout (*Oncorhynchus mykiss*) against infection caused by the bacterium (26). Tricarboxylic acid (TCA) cycle must operate as a complete cycle for *Salmonella enterica serovar Typhimurium SR-11* to be fully virulent but loss of different genes of the TCA cycle causes a different level of loss of virulence (27, 28). Specifically, SR-11 Δ*sucAB* mutant, which is unable to convert α-ketoglutarate to succinyl-coenzyme A (CoA), is avirulent; SR-11 Δ*sucCD* mutant, unable to generate succinate from succinyl-CoA, is moderately attenuated; SR-11 Δ*sdhCDA* mutant, unable to generate fumarate from succinate, is slightly attenuated; and SR-11 Δ*mdh* mutant, unable to convert malate to oxaloacetate, is highly attenuated (27, 28). Thus, altering enzymatic activity may represent an efficient approach to develop live-attenuated vaccines.

Mg^2+^ participates in a multitude of essential processes. The majority of cytosolic Mg^2+^ is involved in various aspects of protein synthesis such as being a cofactor, a counter ion for ATP or neutralizing negative charges from phosphates present in the rRNA and then assemble ribosomes (29, 30). Without sufficient Mg^2+^, ribosomal subunits fall apart and membranes become leaky (31). Free ionic Mg^2+^ regulates many important metabolic enzymes and membrane channels at specific metal binding sites. At concentrations naturally present in seawater, Mg^2+^ improves migration without altering the growth rate of *Vibrio fischeri*. Mg^2+^ addition enhances flagellation, at least in part through an effect on the steady-state levels of flagellin protein (32). The presence of magnesium is a critical factor in promoting type III secretion system of protein substrates in *V. parahaemolyticus* (33). *V. alginolyticus* is a mildly halophilic bacterium which is considered to be *V. parahaemolyticus* type II that can live in seawater with a wide range of magnesium concentrations. Usually, sea water with a salinity of 3.5% contains 54 mM Mg^2+^ (34). However, clinical and nonclinical *V. parahaemolyticus* isolates are resistant to 300 mM Mg^2+^, a concentration that is toxic to many other microorganisms, but bacterial survival is affected (35), suggesting that the high magnesium concentration is a strong stress to these bacteria. Thus, we hypothesized that *V. alginolyticus* cultured in high magnesium concentrations may be a new approach to develop live-attenuated vaccines.

## Materials and Methods

### Bacterial Strain and Culture Conditions

*V. alginolyticus* ATCC33787 was purchased from the Guangdong Province Microbial Culture Collection (GDMCC). *V. alginolyticus* is grown in 3% NaCl 0.5% yeast overnight and 1:100 using fresh 3% NaCl 0.5% yeast medium and grown at 30°C. For growth curve, OD600 of the bacterial cultures was measured in medium with 0, 0.78, 3.125, 12.5, 50 or 200 mM MgCl_2_, which were designed according to the event that 50 mM is approximately the MgCl_2_ concentration of coastal waters and then 50 time or divided by 4 for other concentrations. Notably, different from V12G01 used in a previous report (36), which caused infectious symptoms to significantly appear after 40 h, ATCC33787 led to the similar infectious symptoms after 24 h.

### Swarming and Swimming

*V. alginolyticus* was inoculated into 5 ml of 3% NaCl and 0.5% yeast medium and cultured at 30°C overnight. Then 10 μl of each sample was spotted onto the center of 1.5% or 0.3% agar LB plates with 3% NaCl and 0, 0.78, 3.125, 12.5, 50 or 200 mM MgCl_2_ for swarming and swimming, respectively. These plated samples were incubated in a constant temperature incubator at 30°C for 8 h for the diameter of the halo.

### Fish

Zebrafish (~0.2g body weight), *Danio rerio*, were obtained from a zebrafish breeding corporation (Guangzhou, China). Zebrafish were reared in 25 L water tanks and each tank was equipped with closed recirculating aquaculture systems. These fish were cultured for two weeks before experimental manipulation and were fed twice daily.

### Bacterial Challenge and Sample Preparation for GC–MS Analysis

For the metabolic profile of zebrafish injected by vaccine, zebrafish (n = 180) were randomly divided into two groups, control group (n = 90) and bacterial vaccination group (n = 90). The control group was injected with 5 μl of 3% saline per fish and the vaccination group was intramuscularly injected with 5 μl *V. alginolyticus* cultured with 200 mM MgCl_2_ (2 × 10^5^ CFU) per fish. Spleens were collected at 48 h post-injection. Nine spleens were mixed for a sample. A total of ten samples were obtained for GC–MS analysis.

For the metabolic profile of survival and dying zebrafish from bacterial infection, zebrafish (n = 270) were randomly divided into two groups, control group (n = 90) and bacterial challenge group (n = 180). The control and bacterial challenge groups were intramuscularly injected with 5 μl of 3% saline and 5 μl *V. alginolyticus* (8 × 10^5^ CFU, half lethal dose) per fish, respectively. Spleens were collected at 24 h post-injection. Nine spleens were mixed for a sample. A total of ten samples were obtained for GC–MS analysis.

To prepare the fish sample for the GC–MS analysis, samples were quenched with 1 ml of cold methanol (HPLC, Sigma Aldrich) and sonicated for 5 min at a 200 W power setting. After centrifugation at 12,000*g* for 10 min, 10 μl of 0.1 mg/ml ribitol (Sigma Aldrich) as an analytical internal standard was added. The supernatant was concentrated for 4 h in a rotary vacuum centrifuge device, LABCONCO. The dried polar extracts were used for the GC–MS analysis. Ten biological samples with two technical repeats were separately used for the test and control groups.

### GC–MS Analysis

GC–MS analysis was carried out with a variation on the two stage techniques as described previously (37, 38). In brief, samples were derivatized through two steps. First, 80 μl of 20 mg/ml methoxamine hydrochloride in pyridine (Sigma) was added to the extracts and incubated for 3 h at 37°C. Then, 80 μl N-methyl-N-trimethylsilyltrifluoroacetamide (MSTFA, Sigma-Aldrich) was put and incubated for 1.5 h at 37°C. The samples were centrifuged at 12,000*g* for 10 min at 4°C. A sample analysis was carried out by Agilent 7890A GC equipped with an Agilent 5975C VL MSD detector (Agilent Technologies, Santa Clara, CA, USA). The injector temperature was kept at 270°C, and 0.1 μl aliquot was injected into a column. Temperature program of the GC oven was held at 85°C for 5 min, followed by an increase to 270°C at a rate of 15°C min and then held for 5 min. Helium was used as carrier gas and its flow rate was 1 ml/min. The MS was operated in a range of 50–600 m/z. For each sample, two technical replicates were prepared to confirm the reproducibility of the reported procedures.

### Exogenous Administration of Sodium Malonate and Bacterial Challenge

To investigate the effect of the TCA cycle on host survival, a total of 80 zebrafish were randomly divided into saline group (n = 20) and sodium malonate group (n = 60). For sodium malonate group, the fish were further randomly divided into three subgroups (n = 20). Each subgroup was reared in an individual tank. Control group was injected with 5 μl saline (0.85% sodium chloride) per fish, while the three subgroups were separately injected with 12.5, 25, and 50 mM sodium malonate, once per day for three days *via* intraperitoneal injection. These fish were intramuscularly challenged with 5 μl *V. alginolyticus* (8 × 10^5^ CFU, half lethal dose) per fish.

### Measurement of PDH, a-KGDH, SDH, and MDH Activity

The activity of pyruvate dehydrogenase (PDH), a-ketoglutarate dehydrogenase (a-KGDH), succinate dehydrogenase (SDH) and malate dehydrogenase (MDH) was measured as previously reported with a modification (39). Briefly, visceral organs from three zebrafish were pooled and homogenized in ice-cold PBS (pH 7.4) which were added at a ratio of 1:15 (w/v). The homogenates were centrifuged and protein concentration in supernatant was measured by a BCA Protein Assay Kit (Beyotime, China). The reaction buffer for PDH and a-KGDH included 0.5 mM MTT, 2.5 mM MgCl_2_, 6.5 mM PMS, 0.2 mM TPP, 50 mM PBS, and 2 mM sodium pyruvate (for PDH) or 2 mM sodium α-Ketoglutaric acid (for α-KGDH). The reaction systems of SDH and MDH included 0.5 mM MTT, 13 mM PMS, 50 mM PBS, 5 mM succinate (for SDH) or 50 mM malate (for MDH). All the reactions were performed in a final volume of 200 μl in a 96-well plate. Subsequently, the plate was incubated at 30°C for 10 min for PDH, a-KGDH, SDH, and MDH. The optical absorbance was performed in a microplate reader (Bio-Tek Synergy2, USA) at 566 nm.

### Gene Expression by Quantitative Real-Time Polymerase Chain Reaction (qRT-PCR)

For expression of genes encoding the TCA cycle, zebrafish (n = 252) were divided into five groups, control group (n = 24), vaccination group (n = 24), L group (n = 24), M group (n = 60), and H group (n = 120). Control and vaccination groups were intramuscularly injected with 5 μl saline solution and 5 μl *V. alginolyticus* cultured with 200 mM MgCl_2_ (2 × 10^5^ CFU), respectively, each fish. L group, M group, and H group were intramuscularly injected with 5 μl *V. alginolyticus* (2 × 10^5^ CFU, 8 × 10^5^ CFU, 1.5 × 10^6^ CFU, respectively) each fish. These zebrafish were collected at 24 h post-injection. For expression of genes encoding innate immune immunity, zebrafish were intramuscularly injected with 5 μl (50 or 100 μg) fumaric acid, once a day for three days, and collected from vaccination and bacterial challenge as described earlier. Expression of these genes was analyzed by the qRT-PCR as previously described (39). The total RNA was isolated from spleens pooled from six *D. rerio* with Trizol (Invitrogen, USA). qRT-PCR was performed in 384-well plates with a total volume of 10 μl containing 5 μl 2 × SYBR Premix Ex TaqTM, 4.6 μl H_2_O, 0.1 μl cDNA template and 0.2 μl each of forward and reverse primers (10 mM). The reaction mixtures were run on a LightCycler 480 system (Roche, Germany). Data were shown as the relative mRNA expression compared with the β-actin gene by 2^−ΔΔ^CT method. All qRT-PCR reactions were performed for four biological replicates. Gene-specific primers used for qRT-PCR are shown in **Table 1**.

**Table 1:** Primers used for qPCR analysis.

Gene	Primer	Sequence (5’-3’)	Product size (bp)
β-actin	Forward	ACCCAGACATCAGGGAGTG	112
	Reverse	CATCCCAGTTGGTCACAATAC	
*il1β*	Forward	TGGACTTCGCAGCACAAAATG	139
	Reverse	GTTCACTTCACGCTCTTGGATG	
*il 4*	Forward	TACATTGGTCCCCGTTTCTG	193
	Reverse	ACCCTTCAAAGCCATTCCTG	
*il 8*	Forward	CACGCTGTCGCTGCATTG	127
	Reverse	GTCATCAAGGTGGCAATGATCTC	
*il 21*	Forward	CTAAAGTGCTGCACCTGTCAG	181
	Reverse	TTGCACTGAGCTTTCTGTGTC	
*tnf-α*	Forward	ATAAGACCCAGGGCAATCAAC	177
	Reverse	CAGAGTTGTATCCACCTGTTAAATG	
*c3b*	Forward	TGTGACCCGCTGTATGTTCT	112
	Reverse	TTGGCTGGGAAGTTCTTCAC	
*tlr1*	Forward	CACCTGCGAGGAAAGTAAGT	108
	Reverse	TGTAAGGGCGCAATCAGAC	
*tlr3*	Forward	AGATTCTACACCTGGACATTCTCG	131
	Reverse	CATGATGGGCTTTGAATTG	
*nf-κb*	Forward	GCTCATTCAGATTGCTCTACAC	126
	Reverse	CGTGTCTCCGTTCTCATCT	
*lysozyme*	Forward	GACACTGGGACGCTGTGATG	174
	Reverse	AGGCCGTGCACACATAGTTG	

## Results

### MgCl_2_ Influences Phenotype of *V. alginolyticus*

**Figure 1:**
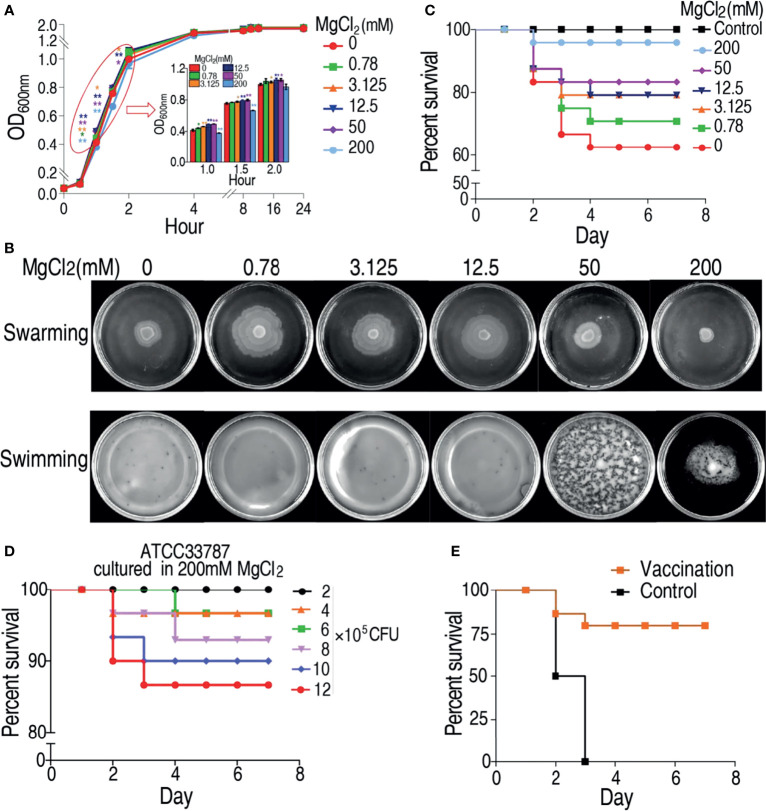
MgCl_2_ regulates ATCC33787 phenotypes. **(A)** Growth curve of ATCC33787 cultured in medium with 0, 0.78, 3.125, 12.5, 50, and 200 mM MgCl_2_.**(B)** Swimming and swarming of ATCC33787 cultured in medium with 0, 0.78, 3.125, 12.5, 50, and 200 mM MgCl_2_. **(C)** Percent survival of zebrafish infected by *V. alginolyticus* cultured in medium with different concentrations of MgCl_2_. **(D)** Percent survival of zebrafish infected by different amounts of *V. alginolyticus* cultured in 200 mM MgCl_2_. **(E)** Percent survival of zebrafish immunized by *V. alginolyticus* cultured in 200 mM MgCl_2_ and challenged by *V. alginolyticus.* Results **(A)** are displayed as mean ± SEM, and significant differences are identified (*p < 0.05; **p < 0.01) as determined by two-tailed Student’s t-test.

To investigate the effect of magnesium ion on physiology of *V. alginolyticus*, growth curve of *V. alginolyticus* ATCC33787 was determined in a gradient concentration of MgCl_2_. Different growth rate of the bacterium was observed in different MgCl_2_ concentrations, especially during the first 1–2 h. The growth rate of ATCC33787 from the fastest to the slowest were in 50 mM ≥ 12.5 mM > 3.125 mM > 0.78 mM > 0 mM > 200 mM MgCl_2_ (**Figure 1A**). Furthermore, swarming and swimming experiments were also carried out in the gradient concentration of MgCl_2_. The strongest swarming was measured in plates with 0.78 mM MgCl_2_ and then reduced with the increasing MgCl_2_ (**Figure 1B**). Interestingly, the swarming ability was lower in plate with 200 mM MgCl_2_ than without MgCl_2_ (**Figure 1B**). However, MgCl_2_ concentration did not affect swimming ability in plates with 0.78–50 mM MgCl_2_ and without MgCl_2_, where the bacterium diffused to all the plates but not evenly distributed in 50 mM MgCl_2_. When the concentration of MgCl_2_ reached 200 mM, the distribution was depressed (**Figure 1B**). Importantly, virulence of ATCC33787 to zebrafish was reduced with the increasing MgCl_2_ concentrations, where similar survival was detected in zebrafish infected by bacteria cultured in 200 mM MgCl_2_ and saline control (**Figure 1C**). When zebrafish were infected with different numbers of 200 mM MgCl_2_-cultured bacteria, percent survival of zebrafish was elevated with the decreasing numbers of bacteria. Among them, all animals were survived at the infection dose of 2 × 10^5^ CFU bacteria (**Figure 1D**). Furthermore, zebrafish were immunized with 2 × 10^5^ CFU of 200 mM MgCl_2_-cultured bacteria or saline. Then, zebrafish were challenged with a lethal dose of 5 × 10^6^ CFU *V. alginolyticus* ATCC33787. When all animals died in the control group, 76% animals survived in the experiment group (**Figure 1E**). These results indicate that 200 mM MgCl_2_-cultured ATCC33787 can be used as a live-attenuated vaccine candidate against infection caused by *V. alginolyticus*.

### Metabolome of Zebrafish Immunized With the Live-Attenuated Vaccine Candidate

**Figure 2:**
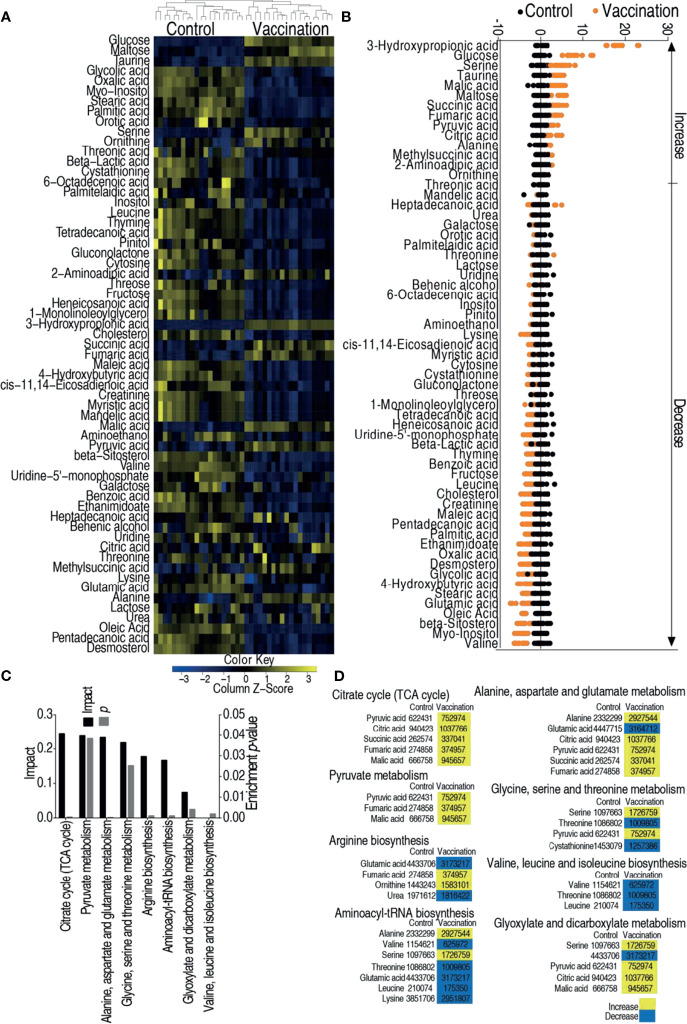
Metabolomic analysis of zebrafish to *V. alginolyticus* cultured in 200 mM magnesium. **(A)** Heat map showing relative abundance of metabolites (Wilcoxon P <0.05) in control, vaccination groups. Heat map scale (blue to yellow: low to high abundance) is shown at bottom. **(B)** Z scores map in control, vaccination groups. **(C)** Enriched metabolic pathways in control, vaccination groups. **(D)** Change of the abundance of the metabolites. The lists of the 60 metabolites enriched in the eight pathways in panel **(C)**. Yellow indicates increase; Blue indicates decrease.

Hosts against bacterial infection have infective and anti-infective metabolomes, which decide the consequences of infection (36, 40). This motivated us to explore the metabolic mechanisms by which the vaccine candidate protects zebrafish against the bacterial infection. To test this idea, GC–MS based metabolomics was adopted to investigate the metabolic profiles of control group (injected with saline) and vaccination group (injected with 2 × 10^5^ CFU of the *V. alginolyticus* vaccine). Nine spleens were pooled as one sample. A total of 240 aligned peaks were identified in each sample. After the removal of internal standard, ribitol, any known artificial peaks, and merge of the same compounds, 80 metabolites with reliable signals were identified in each sample. Ten samples with two technical repeats for each sample were included in each group, yielding 40 data sets. The correlation coefficient between technical replicates varied between 0.996 and 0.999, demonstrating the reproducibility of the data (**Supplementary Figure 1A**). According to the Kyoto Encyclopedia of Genes and Genomes (KEGG), 38, 27, 20, 12, and 3% of the metabolites were categorized to carbohydrate, amino acid, fatty acid, nucleotide, and others, respectively (**Supplementary Figure 1B**). The metabolomic profiles of these two groups were displayed as heat map (**Supplementary Figure 1C**). Kruskal–Wallis test was used to compare the two groups, where 61 differential abundances of metabolites were identified as shown in **Figure 2A**. A Z-score plot ranged from −4.9 to 18.1 in the vaccination group compared to control group and showed 15 increased metabolites and 46 decreased metabolites (**Figure 2B**). Among these differential abundances of metabolites, 37, 34, 17, and 12% of the metabolites were carbohydrate, amino acid, lipid, and nucleotide (**Supplementary Figure 2**). These differential abundances of metabolites were analyzed and outlined in KEGG (http://www.genome.jp/kegg) and MetPA (http://metpa.metabolomics.ca), respectively, for pathway enrichment. A total of eight metabolic pathways were enriched, where the top three impactful pathways were TCA cycle, pyruvate metabolism, alanine, aspartate and glutamate metabolism (**Figure 2C**). Interestingly, among the eight enriched pathways, all differential abundances of metabolites were elevated in the TCA cycle and pyruvate metabolism but reduced in valine, leucine, and isoleucine biosynthesis (**Figure 2D**). Therefore, the TCA cycle can be further explored for vaccine-conferred protection.

### Metabolome of Zebrafish Infected With LD50 of ATCC33787

**Figure 3:**
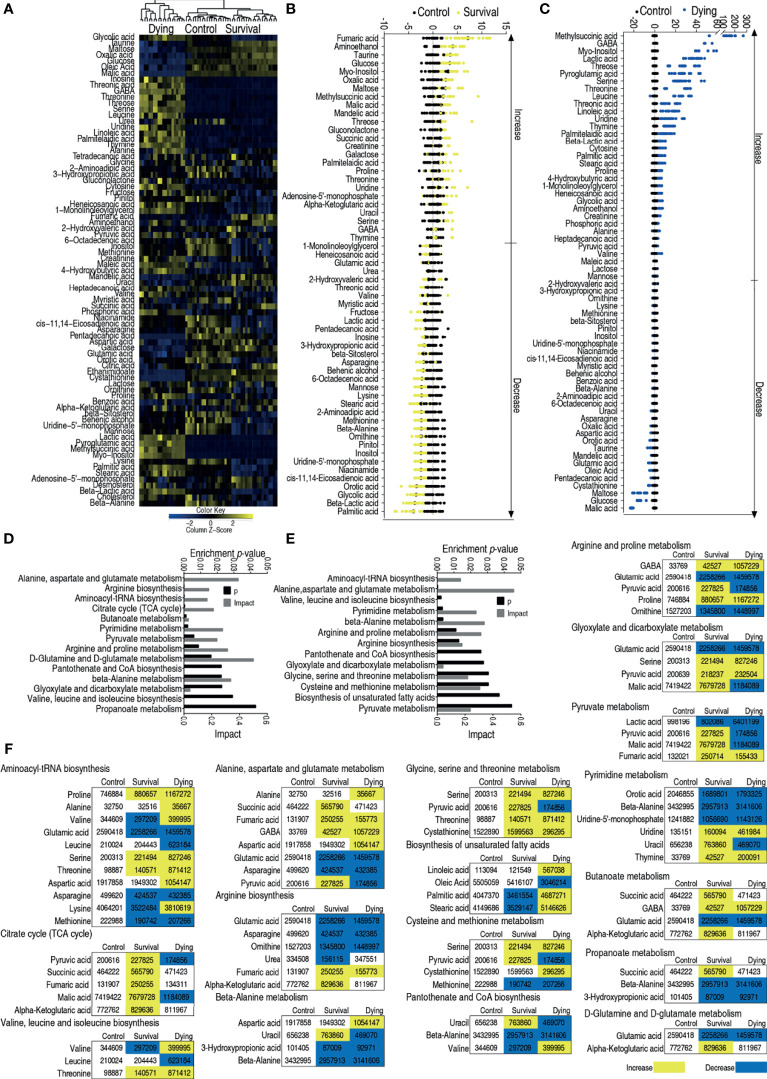
Enriched pathways between dying and survival groups. **(A)** Heat map showing relative abundance of metabolites in control, survival and dying groups. Heat map scale (blue to yellow: low to high abundance) is shown at bottom. **(B)** Z scores map in control and survival groups. **(C)** Z scores map in control and dying groups. **(D)** Enriched metabolic pathways in control and survival groups. **(E)** Enriched metabolic pathways in control and dying groups. **(F)** Change of the abundance of the metabolites. Yellow indicates increase, Blue indicates decrease.

To understand differential metabolic profiles between survival and dead zebrafish infected with *V. alginolyticus*, GC–MS was used to characterize the metabolomes of survival and dying zebrafish infected with LD50 of ATCC33787 and saline group. GC–MS was performed as described above. The identified 72 metabolites (p <0.05) in survival and dying groups were shown as heatmap, where survival and control groups were clustered (**Figure 3A**). The differential abundances of metabolites were identified between the control and survival groups or control and dying groups by Kruskal–Wallis test. Approximately 59 metabolites and 64 metabolites were identified (p <0.05) in survival metabolome and dying metabolome, respectively. Z scores value of these differential metabolites showed that fumaric acid was the most upregulated metabolite in the survival group, and malic acid was the most downregulated substance in the death group (**Figures 3B,C**). Pathway analysis of 59 differential metabolites in survival group and 64 differential metabolites in dying group were performed in MetPA, fourteen and thirteen pathways were enriched, respectively, as shown in **Figures 3D, E**. Interestingly, all metabolites of the TCA cycle and glycine, serine and threonine metabolism were elevated (**Figure 3F**). These results indicate that the elevated TCA cycle and glycine, serine and threonine metabolism are required for the survival.

### Shared Metabolic Modulation Between the Vaccination and Infection

**Figure 4:**
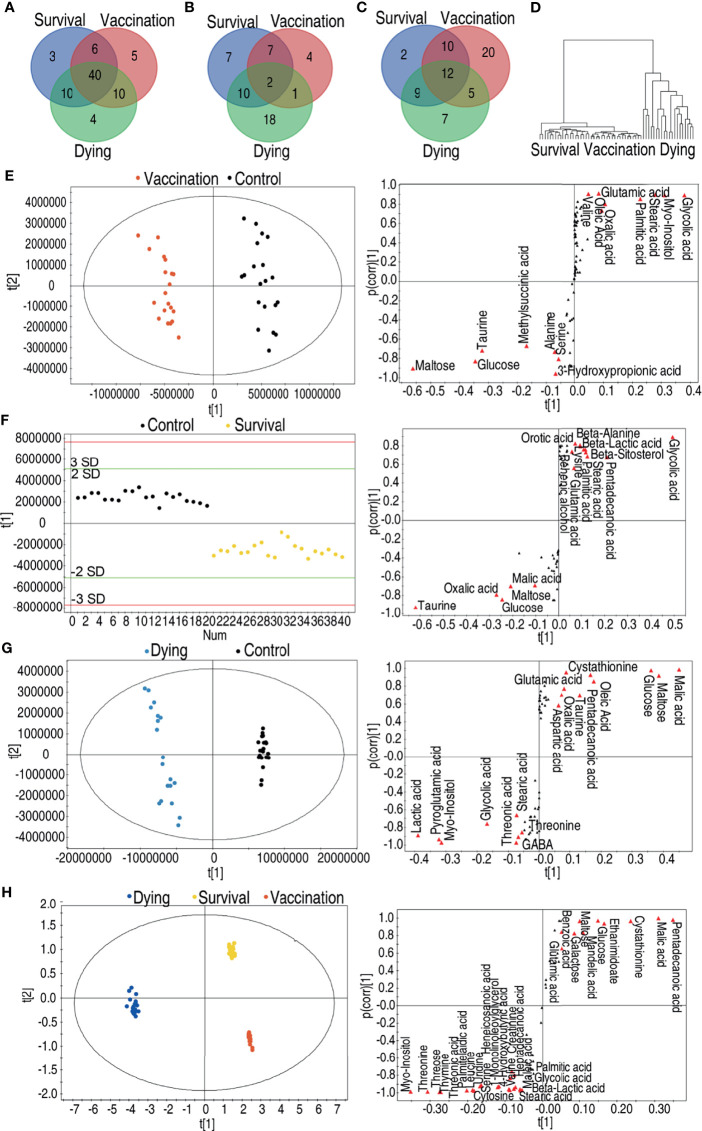
Comparative analyses of vaccination group, survival group and dying group. **(A)** Venn diagram of the total differential metabolites between the vaccination group, the survival group and the dying group. **(B)** Venn diagram of the upregulated differential metabolites between the vaccination group, the survival group and the dying group. **(C)** Venn diagram of the downregulated differential metabolites between the vaccination group, the survival group and the dying group. **(D)** Clusters of global substances’ relative changes between vaccination group, survival group and dying group. **(E)** Scores plot and S-plot of OPLS-DA model between control, vaccination group in data (**Figure 2A**). Red triangles highlight candidate biomarkers. **(F)** Scores plot and S-plot of OPLS-DA model between control and survival group in data (**Figure 3A**). Red triangles highlight candidate biomarkers. **(G)** Scores plot and S-plot of OPLS-DA model between control and dying group in data (**Figures 2A** and **3A**). Red triangles highlight candidate biomarkers. **(H)** Scores plot and S-plot of OPLS-DA model between vaccination, survival and dying group in data. Red triangles highlight candidate biomarkers.

It is interesting to compare the metabolomes induced by the vaccine and mediated by the infection. To do this, a Venn diagram was used to exhibit overlapped and unique metabolites among 61, 59, and 64 differential abundances of metabolites of the vaccination group, survival group, and dying group, respectively. Forty metabolites were shared among these three groups; 6, 10, and 10 were overlapped between the survival and vaccination groups, survival and dying groups, vaccination and dying groups, respectively; 3, 5, and 4 existed only in the survival, vaccination and dying groups, respectively (**Figure 4A**). Among these 14, 26, and 31 upregulated metabolites of the vaccination, survival and dying groups, respectively, 2 metabolites were shared by the three groups; 7, 10, and 1 metabolites were shared by the survival and vaccination groups, survival and dying groups, vaccination and dying groups, respectively; 7, 4, and 18 metabolites were only present in the survival, vaccination and dying groups, respectively (**Figure 4B**). Among the 47, 33, and 33 downregulated metabolites of the vaccination, survival and dying groups, respectively, 12 metabolites were shared by the three groups; 10, 9, and 5 were shared by the survival and vaccination group, survival and dying group, vaccination and dying group respectively; 2, 20, and 7 were only present in the survival, vaccination and dying groups, respectively (**Figure 4C**). When all of the identified metabolites were analyzed together among the three groups, the vaccination and survival groups were clustered (**Figure 4D**), indicating the survival and vaccination groups have similar metabolic profiles.

Moreover, orthogonal partial least squares discriminant analysis (OPLS-DA) was adopted to identify shared biomarkers between vaccination and survival groups. To do this, S-plot was used for identification of discriminatory variables. Cut-off values were set as greater or equal to 0.5 and 0.05 absolute value of covariance *t* and correlation *p* (corr), respectively. As compared to the control group, seven metabolites were increased and eight metabolites were decreased in the vaccination group (**Figure 4E**). Whereas five metabolites were increased and 10 metabolites were decreased in the survival group (**Figure 4F**), while eight metabolites were increased and 10 metabolites were decreased in the dying group (**Figure 4G**). Among the shared metabolites, the abundance of maltose, glucose, taurine was upregulated and the abundance of glycolic acid, myo-inositol, stearic acid, palmitic acid was downregulated in both of the survival and vaccination groups. Malic acid, an intermediate metabolite of the TCA cycle, was increased in the survival group and decreased in the dying group (**Figures 4E**–**G**). PCA analysis among the survival group, dying group and vaccination group further confirms this conclusion. Compared to the dying group, pentadecanoic acid, malic acid, cystathionine, ethanimidoate, glucose, mandelic acid, maltose, galactose, benzoic acid and glutamic acid were increased; myo-inositol, threonine, threonic acid, thymine, threose, palmitelaidic acid, leucine, serine, uridine, heneicosanoic acid, 1-monolinoleoyllglycerol, 4-hydroxybutyric acid, valine, creatinine, heptadecanoic acid, maleic acid, cytosine, stearic acid, palmitic acid, glycolic acid and beta-lactic acid were decreased in the survival and vaccination groups (**Figure 4H**). These results indicate that the metabolic flux of glycolysis to the TCA cycle instead of fatty acid biosynthesis is shared by the vaccination and survival groups.

### Elevation of the TCA Cycle Is Required for Vaccine Efficacy and Survival From Infection

**Figure 5:**
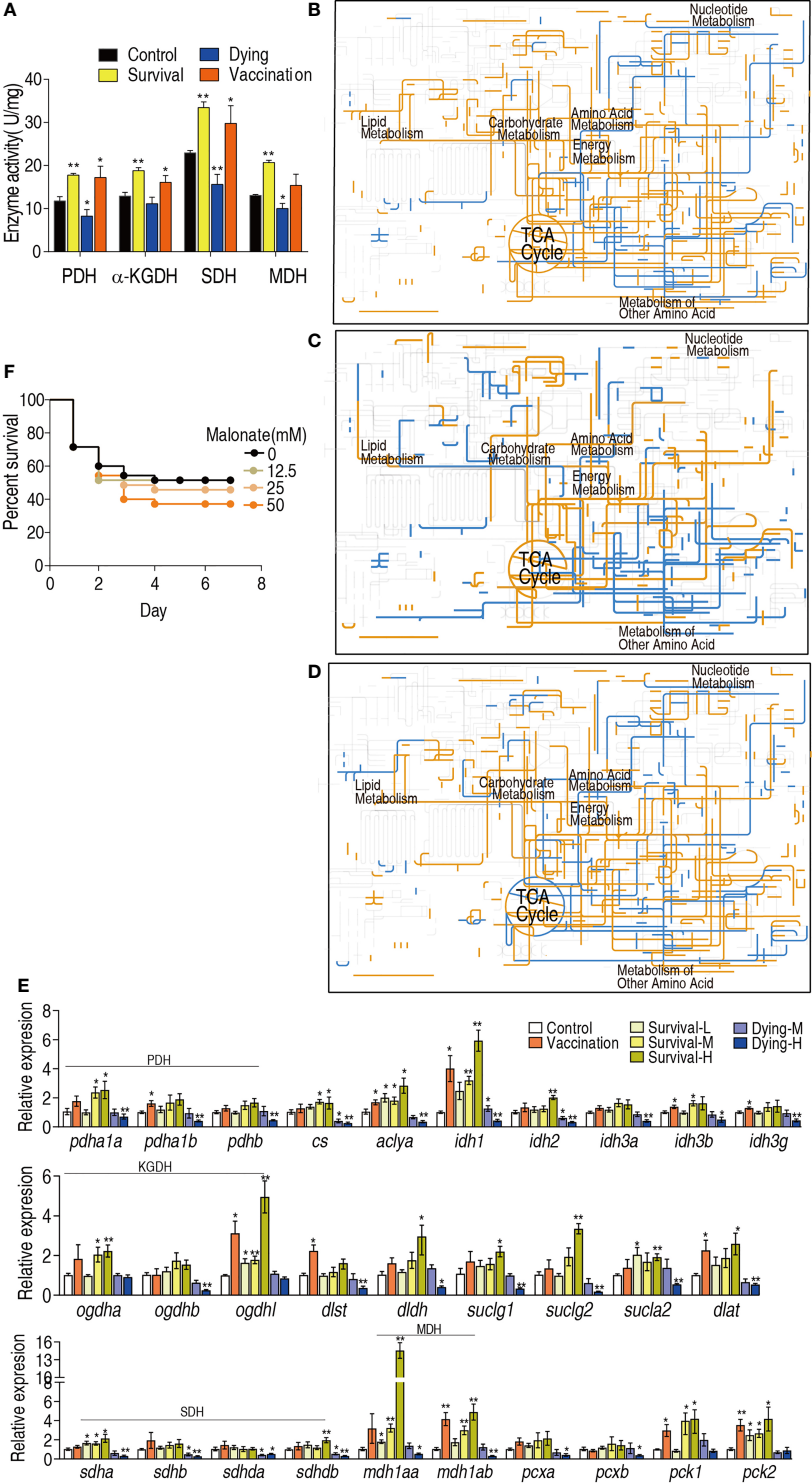
iPath analysis and activity of enzymes in dying and survival groups. **(A)** Enzyme activity of TCA cycle. **(B)** iPath analysis of the metabolites of differential abundance in the survival group. **(C)** iPath analysis of the metabolites of differential abundance in the vaccination group. **(D)** iPath analysis of the metabolites of differential abundance in the dying group. **(E)** qRT-PCR for expression of genes encoding the TCA cycle of zebrafish challenged by high (1.5 × 10^6^ CFU, H), middle (8 × 10^5^ CFU, M) and low (2 × 10^5^ CFU, L) doses of ATCC33787 and the 200 mM MgCl_2_-prepared live-attenuated vaccine (vaccination) and saline solution was used control. **(F)** Percentage of survival of *D. rerio* when TCA cycle was blocked by inhibitor. Results **(A, E)** are displayed as mean ± SEM, and significant differences are identified (*p < 0.05; **p < 0.01) as determined by two-tailed Student’s t-test.

The above results suggest that the TCA cycle plays a crucial role in the protection against bacterial infection. To confirm this, the activity of pyruvate dehydrogenase (PDH), α-Ketoglutarate dehydrogenase (KGDH), succinate dehydrogenase (SDH) and malate dehydrogenase (MDH) in the TCA cycle and pyruvate metabolism were measured. The activity of all enzymes was elevated in the vaccination group, survival group and reduced in the dying group (**Figure 5A**). Then, iPath was used to compare metabolic pathways among the vaccination, survival and dying groups. The resulting global overview map provided a better insight into the effects of vaccination and infection consequence on the metabolism of the fish, where yellow and blue lines represented increased and decreased pathways in the reprogramming group, respectively. Elevation of the TCA cycle in the vaccination and survival groups and fluctuation of the TCA cycle in dying group form the most characteristic feature (**Figures 5B**–**D**). Furthermore, qRT-PCR was used to detect expression of genes encoding the TCA cycle of zebrafish challenged by high (1.5 × 10^6^ CFU, H), middle (8 × 10^5^ CFU, M) and low (2 × 10^5^ CFU, L) doses of ATCC33787 and the 200 mM MgCl_2_-prepared live-attenuated vaccine (vaccination) and saline solution was used control. They caused 15% survival (survival-H) and 85% dying (dying-H), 60% survival (survival-M) and 40% dying (dying-M), 100% survival (survival-L), and 100% survival (vaccination), respectively. On the whole, higher expression of genes was detected in the three survival groups and vaccination group than the control, while lower expression of genes was measured in the two dying groups than the control. The high expression was ranked as survival-H > survival-M and vaccination > survival-L and low expression was listed as dying-H > dying-M (**Figure 5E**). Finally, sodium malonate, an inhibitor of SDH, decreased the survival of zebrafish infected with ATCC33787 in a dose-dependent manner (**Figure 5F**). These results indicate that elevation of the TCA cycle is required for the survival of zebrafish infected with ATCC33787. Consistently, the elevated TCA cycle is a possible reason why the live-attenuated vaccine provides an effective ability against the infection by the bacterium.

### Vaccine Enhances Innate Immune Response

**Figure 6:**
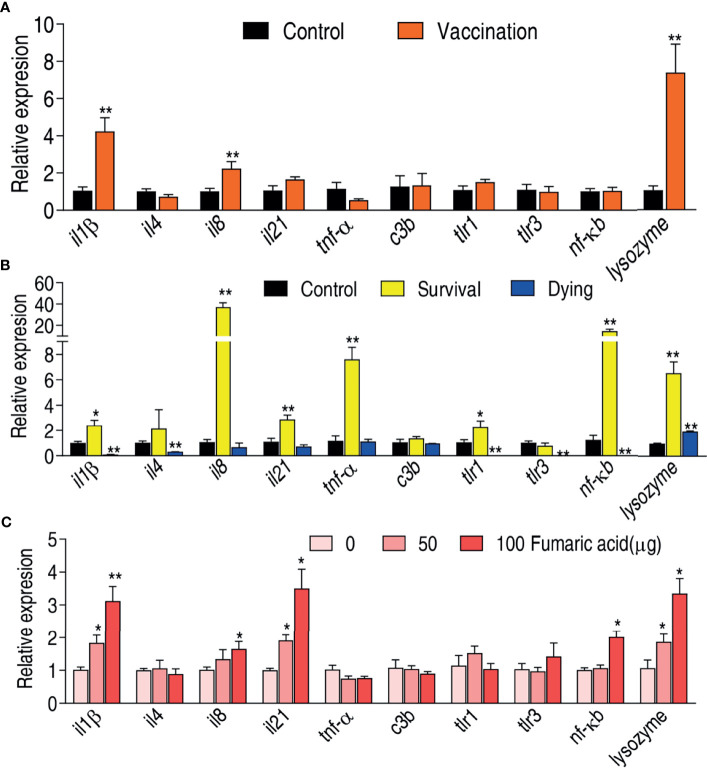
Immune response of zebrafish to *V. alginolyticus.* **(A)** qRT-PCR for quantifying transcriptional levels of innate immune genes in zebrafish immunized by vaccine. **(B)** qRT-PCR for quantifying transcriptional levels of innate immune genes in zebrafish of survival and dying groups. **(C)** qRT-PCR for quantifying transcriptional levels of innate immune genes in zebrafish after injected by fumaric acid. Results are displayed as mean ± SEM, and significant differences are identified (*p < 0.05; **p < 0.01) as determined by two-tailed Student’s t-test.

Innate immune response contributes to host immune protection against bacterial infection (41, 42). Therefore, it is required to investigate whether the live-attenuated vaccine activates an innate immune response. The expression of ten innate immune genes *il1β*, *il4*, *il8*, *il21*, *tnf-α*, *c3b, tlr1*, *tlr3*, *nf-κb*, and *lysozyme* were measured. Compared to the saline control, the vaccination group exhibited elevated expression of *il1β*, *il8*, and *lysozyme* but the rest of the genes remain unaffected (**Figure 6A**). Meanwhile, expression of these genes was compared between the survival group and the dying group. The expression of *il1β*, *il8*, *il21*, *tnf-α*, *tlr1*, *nf-κb*, and *lysozyme* was elevated in the survival group, while the expression of *il1β, il4*, *tlr1*, *tlr3*, and *nf-κb* was reduced in the dying group (**Figure 6B**). Importantly, the elevated *il1β*, *il8*, and *lysozyme* were shared between the vaccination and survival groups, where expression of *il1β* was reduced in the dying group.

Recently, we have showed that malic acid of the TCA cycle promotes expression of innate immune genes and thereby improves the survival of zebrafish infected with *V. alginolyticus* (40). We speculate that the elevated TCA cycle is related to the elevated expression of innate immune genes. To test this, fumaric acid of the TCA cycle was used to test whether it can promote expression of the three shared genes *il1β*, *il8*, and *lysozyme*. Indeed, exogenous fumaric acid increased expression of *il1β*, *il8*, and *lysozyme* in a dose-dependent manner. Besides these, the metabolite also promoted the expression of *il21* and *nf-κb* (**Figure 6C**). These results indicate that the live-attenuated vaccine improves the immune level of zebrafish by promoting the TCA cycle, and thus elevates the survival rate of zebrafish infected with *V. alginolyticus*.

## Discussion

Reports have indicated that targeting at metabolic enzymes is an efficient way to develop live-attenuated vaccines (26, 27). Mg^2+^ participates in a multitude of essential processes as a cofactor of enzymes (29, 30). The present study explored the effect of different concentrations of MgCl_2_ on bacterial virulence to zebrafish, and showed that *V. alginolyticus* cultured in 200 mM MgCl_2_ were most potent in reducing virulence, including avirulence to zebrafish and a limited flagellar movement, which is also related to bacterial virulence (43). However, the 200 mM MgCl_2_-cultured *V. alginolyticus* still kept immune protection against *V. alginolyticus* infection and thereby can be used as a live-attenuated vaccine candidate. Furthermore, we explored the mechanisms by which the live-attenuated vaccine protects fish to be free of bacterial infection *via* metabolic modulation. To do this, comparison among the vaccine-induced metabolome and bacterial infection-mediated survival metabolome and dying metabolome was performed to identify shared characteristic features in metabolism. Pieces of evidence indicated that the activation of the TCA cycle is required for zebrafish to survive from *V. alginolyticus* infection, where a key metabolite, fumarate, identified from the shared characteristic feature can promote innate immune response. Therefore, the present study develops a previously unreported approach to study mechanisms by which vaccines provide immune protection, which highlights a way in vaccine design and exploration of the underlying mechanisms.

The core finding of the present study is that the live-attenuated vaccine modulates metabolism to regulate innate immune response. Recently, a line of evidence has indicated that there is a close relationship between immune response and metabolism (44–48), but that vaccines activate an innate immune response against bacterial challenge *via* modulating metabolism is unknown. The enhancement of the TCA cycle forms a characteristic feature when immunized with the live-attenuated vaccine, which is consistent with the elevated and decreased expression of genes encoding the TCA cycle in survival and dying groups, respectively. To validate whether the metabolic modulation is related to immune protection, the present study demonstrated that the enhancement of the TCA cycle is an indicator that indicates vaccine efficacy against infection with *V. alginolyticus*. On the contrary, the inactivation of the TCA cycle was identified in zebrafish that died of infection. This interesting finding was further confirmed by an inhibitor sodium malonate of the TCA cycle. These results indicate that the activation of the TCA cycle can protect zebrafish against infection caused by *V. alginolyticus*, which is a mechanism by which the *V. alginolyticus* cultured in 200 mM MgCl_2_ provides immune protection. Yang et al. found that boosted TCA cycle enhanced the survival of zebrafish to *V. alginolyticus* infection, which may be attributed to providing increased immunity against the infection (36, 40). Gong et al. found that the inhibition of pyruvate metabolism and TCA cycle decreased *D. reiro* survival against *V. alginolyticus* (43). Therefore, the TCA cycle is key to provide protection against *V. alginolyticus* infection.

The present study further used fumarate, an intermediate metabolite of the TCA cycle, to explore why the activated TCA cycle can promote immune protection against the infection. In total, four intermediate metabolites of the TCA cycle, namely, fumarate, succinate, malate, and *α*-ketoglutarate, were detected in the GC–MS analysis. Among them, the abundance of fumarate ranked from high to low: survival from the infection > vaccination > dying from the infection group, which is no different with the control group. Thus, fumarate was selected. Exogenous fumarate promoted the expression of *il1β*, *il8*, *il21*, *nf-κb*, and *lysozyme* in a dose-dependent manner. Among the five innate immune genes, the elevated *il1β*, *il8*, and *lysozyme* were overlapped in the vaccine-immunized zebrafish and the survival from the infection. IL-1β and IL-8 are pro-inflammatory cytokines that mediate different kinds of immune responses (44), while lysozyme catalyzes the destruction of the cell walls of certain bacteria (44, 49). They play a key role in the innate immunity against invaded bacterial pathogens. Thus, the activated TCA cycle increased fumarate, thus promoting innate immunity. This finding is consistent with the recent reports that metabolites regulate innate immune response (43, 50, 51).

In summary, the present study develops a previously unreported approach to prepare a live-attenuated *V. alginolyticus* vaccine by culturing bacteria in a high concentration of magnesium to attenuate bacterial virulence. Furthermore, the mechanisms of the live-attenuated vaccine are explored through understanding metabolic modulation. It is revealed that the live-attenuated vaccine activates the TCA cycle and thereby elevated intermediated metabolites such as fumarate to regulate innate immunity. In our knowledge, this is first report to clarify vaccine-induced mechanisms by metabolic modulation.

## Data Availability Statement

The original contributions presented in the study are included in the article/**Supplementary Material**. Further inquiries can be directed to the corresponding author.

## Ethics Statement

The animal study was reviewed and approved by the Institutional Animal Care and Use Committee of Sun Yat-sen University (Approval No. SYSU-IACUC-2020-B126716).

## Author Contributions

HL and X-XP conceptualized and designed the project. JY, X-LY and Y-BS performed experiments. JY and HL interpreted the data. HL and X-XP wrote the manuscript. All authors contributed to the article and approved the submitted version.

## Funding

This work was sponsored by the Projects of Natural Science Foundation of China for International Cooperation and Exchanges NSFC (32061133007, 31911530183) and the Innovation Group Project of Southern Marine Science and Engineering Guangdong Laboratory (Zhuhai) (311021006).

## Conflict of Interest

The authors declare that the research was conducted in the absence of any commercial or financial relationships that could be construed as a potential conflict of interest.

## Publisher’s Note

All claims expressed in this article are solely those of the authors and do not necessarily represent those of their affiliated organizations, or those of the publisher, the editors and the reviewers. Any product that may be evaluated in this article, or claim that may be made by its manufacturer, is not guaranteed or endorsed by the publisher.

## Supplementary Material

Supplementary Figure 1 Metabolomic profiling of vaccinated *D. rerio* speen. **(A)** Reproducibility of metabolomic profiling platform. Metabolite abundances quantified in cell samples over two technical replicates are shown. Correlation coefficient between technical replicates varies between 0.991 and 0.999. This plot shows the two replicates with the weakest correlation of 0.991. **(B)** Categories of the different metabolites. Eighty metabolites with different abundance were searched against in KEGG for their categories. **(C)** Heat map showing relative abundance of eighty metabolites in control and vaccination groups. Heat map scale (blue to yellow: low to high abundance).

Supplementary Figure 2 Categories of the 61 differential metabolites.
