# Supplementary material for: Activation of the TCA Cycle to Provide Immune Protection in Zebrafish Immunized by High Magnesium-Prepared Vibrio alginolyticus Vaccine
Source: Front Immunol. 2021 Dec 7;12:739591. doi: 10.3389/fimmu.2021.739591 (PMC8688852; doi:10.3389/fimmu.2021.739591)
Supplement: Supplementary Figure 1 — Metabolomic profiling of vaccinated D. rerio speen. (A) Reproducibility of metabolomic profiling platform. Metabolite abundances quantified in cell samples over two technical replicates are shown. Correlation coefficient between technical replicates varies between 0.991 and 0.999. This plot shows the two replicates with the weakest correlation of 0.991. (B) Categories of the different metabolites. Eighty metabolites with different abundance were searched against in KEGG for their categories. (C) Heat map showing relative abundance of eighty metabolites in control and vaccination groups. Heat map scale (blue to yellow: low to high abundance). [file DataSheet_1.pdf]

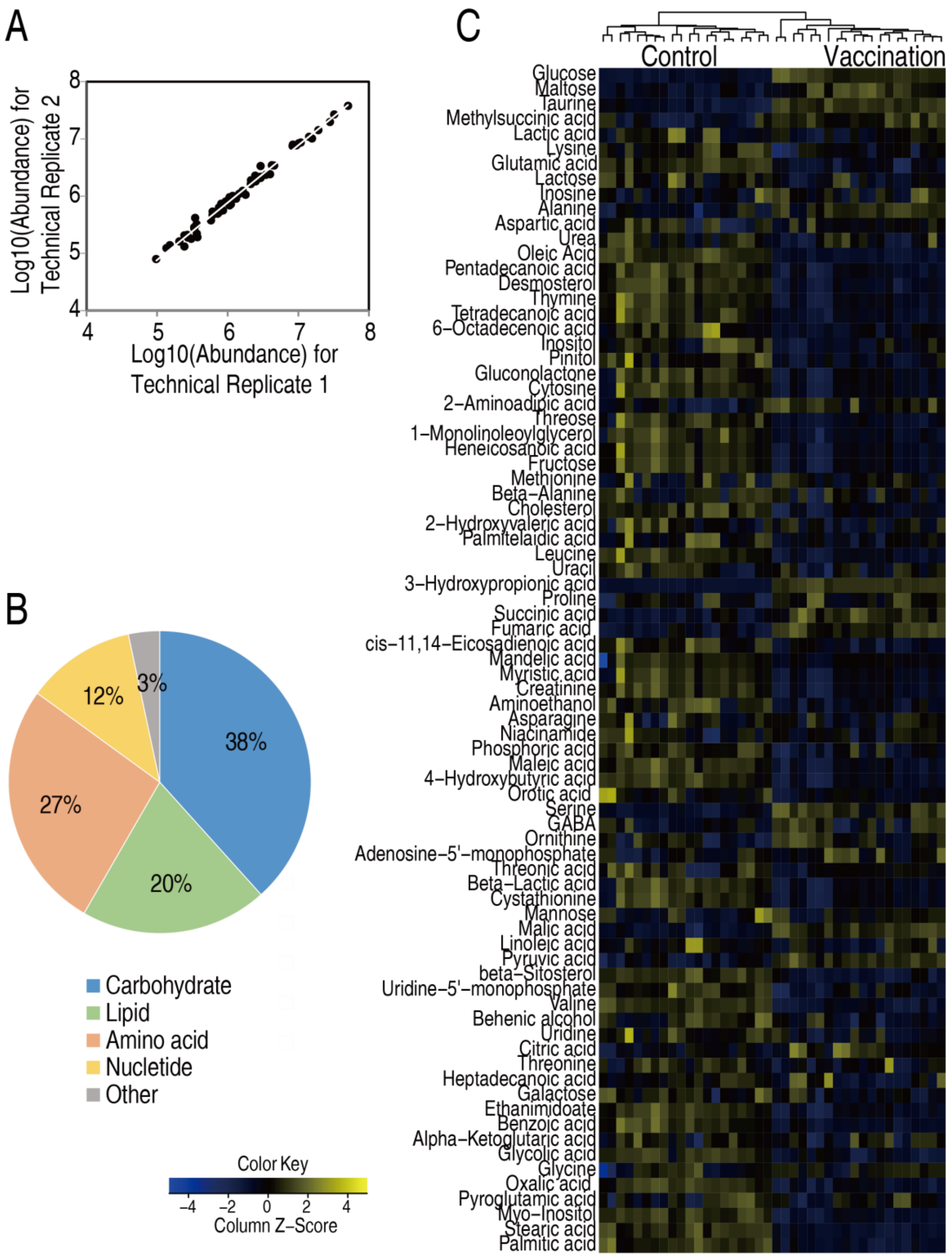

Supplementary Figure.1 Metabolomic profiling of vaccinated *D. rerio* speen. (**A**) Reproducibility of metabolomic profiling platform. Metabolite abundances quantified in cell samples over two technical replicates are shown. Correlation coefficient between technical replicates varies between 0.991 and 0.999. This plot shows the two replicates with the weakest correlation of 0.991. (**B**) Categories of the different metabolites. Eighty metabolites with different abundance were searched against in KEGG for their categories. (**C**) Heat map showing relative abundance of eighty metabolites in control and vaccination groups. Heat map scale (blue to yellow: low to

high abundance).

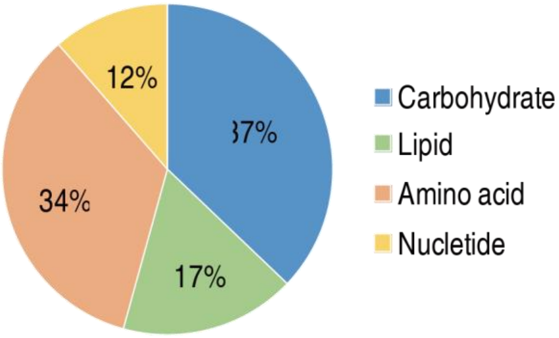

Supplementary Figure.2 Categories of the 61 differential metabolites.
